# *Math5* promotes retinal ganglion cell expression patterns in retinal progenitor cells

**Published:** 2007-06-30

**Authors:** Jing Yao, Xinghuai Sun, Yang Wang, Gezhi Xu, Jiang Qian

**Affiliations:** 1 Ophthalmology, the Affiliated Eye and ENT Hospital, Shanghai Medical School, Fudan University, Shanghai, China; 2 Department of Anatomy and Embryo, Shanghai Medical School, Fudan University, Shanghai, China

## Abstract

**Purpose:**

To investigate the role of over-expression of *Math5* on the retinal ganglion cell (RGC) expression patterns in retinal progenitor cells (RPCs).

**Methods:**

RPCs were cultured and then transfected by recombinant *Math5* plasmid with internal ribosome entry site and enhanced green fluorescent protein (pIRES2-EGFP-*Math5*; group A), with pIRES2-EGFP transfected (group B) and no plasmid transfected (group C) as control. RGCs were identified by Thy1.1 immunocytochemistry methods and analyzed by Leica Qwin V3.1 system. Real-time polymerase chain reaction was used to examine the expression of *Math5*-associated genes at different time points during the differentiation of RPCs.

**Results:**

It was determined that pIRES2-EGFP-*Math5* could transfect RPCs, and the transfection rate was 24.68%. After plating, it was found that three different groups of RPCs could differentiate and express retina-specific markers, including RGC marker Thy1.1. The percentage breakdown of Thy1.1-positive cells was 30.85±6.28% in group A, 15.84±3.55% in group B, and 16.22±3.60% in group C. The differences between the three groups were statistically significant (p<0.001). Transfection by pIRES2-EGFP-*Math5* could change the expression of Delta-1, Hes1, and Brn-3b.

**Conclusions:**

*Math5* may up-regulate RGC expression patterns in RPCs and change the expression of *Math5*-associated genes.

## Introduction

Progressive death of retinal ganglion cells (RGCs) is the cause of debilitating visual impairment associated with prevalent ocular diseases. RGC death results in irreversible functional deficits, and at this point, there is no effective therapy, although stem cell-replacement therapy may be a possible approach [1]. Previous studies have shown that neural stem/progenitor cells are difficult to differentiate into mature retinal cells and that they fail to express any retina-specific markers [2,3]. However, in some special environments, retinal progenitor cells (RPCs) are able to differentiate into mature retinal cells, including RGCs [4], but the percentage of RGCs generated is too low to replace the damaged cells. Increasing the percentage of RGCs would be useful in future cell-replacement therapy.

A cell differentiation model demonstrates that retinal cell specification is determined by intrinsic limitations and the exterior microenvironment [5]. The regulating network involved in RPCs differentiating into RGCs is complex, and the intrinsic factors, especially the different expression of the basic helix-loop-helix (bHLH) transcription factors, appear to play an important role [5,6].The bHLH family includes proneural genes such as atonal homologs (Ath) and achaetescute homologs (Ash) and anti-neural genes such as the Id group and Hes group [5]. Previous studies have shown that *Math5*, a murine bHLH gene, which belongs to the Ath group, is essential for enabling RPCs to differentiate into RGCs [7,8], but the mechanism by which it does so is unknown. In order to investigate their differentiation especially into RGCs, we employed plasmid transfection in this study to make RPCs over-express *Math5*. We then examined the expression of *Math5*-associated genes at different time points during the differentiation of RPCs.

## Methods

### Cell culture

120 E14 Sprague Dawley rats (SLAC Laboratory Animal Co. Ltd., Shanghai, P.R.China) were handled in accordance with the Association for Research in Vision and Ophthalmology statement for the Use of Animal in Ophthalmic and Vision Research. Retinas, without retinal pigment epithelial cells, were carefully removed, incubated in D-hanks (Ca^2+^ and Mg^2+^ free) containing 0.05% trypsin, 1 mM EDTA and 0.1 mg/ml *DNase*1 and gently shaken at 37 °C for 10 min. Trypsin, EDTA, DNase1, and trypsin inhibitor used in this study were from Sino-American Biotechnology Company, Shanghai, P.R.China. Trypsin was neutralized by washing the tissue in 0.25% trypsin inhibitor. Cells were centrifuged at 1000 rpm for 5 min and the supernatant removed. Cells were then re-suspended in DMEM/F12 medium containing 4 mM L-glutamine (Gibco, Carlsbad, CA), 1x N2 supplement (Gibco), 1x B27 supplement (Gibco), 20 ng/ml of basic fibroblast growth factor (bFGF; Sigma, St Louis, MO), and 20 ng/ml of epidermal growth factor (EGF; Sigma). Next, cells were plated into uncoated T25 flasks at a density of approximately 1x10^6^ cells/ml and grown at 37 °C in 95% humidity and 5% CO_2_. Cells were passaged at 1:2 every 5-7 days. All experiments were performed at passage 2-4. In some experiments, 10 μM 5-bromo-2'-deoxyuridine (BrdU, Sigma) was added for the last 24 h to label proliferating cells.

### Plasmid construction

The cDNA fragment encoding math5 protein was obtained from pCS2-Math5 (generously gifted by Dr. N.L. Brown) by polymerase chain reaction (PCR) amplification. The plasmid with internal ribosome entry site (IRES) and enhanced green fluorescent protein (EGFP; pIRES2-EGFP; BD Biosciences Clontech, Mountain View, CA) used in this study could express green fluorescent protein as an internal control for protein expression. Then the cDNA fragment was ligated and subcloned into the *Sal* I and *Bam*H I sites of pIRES2-EGFP vector to create pIRES2-EGFP-*Math5*. The oligonucleotide primer pair used for PCR amplification was as follows: 5'-GCG TCG ACA TGA AGT CGG CCT GCA AAC-3' and 5'-GCG GAT CCT TAG CTG GCC ATG GGG AAG-3'. The amplification was performed using the following cycling protocol: initial denaturation at 94 °C for 5 min, 25 cycles with denaturation at 94 °C for 30 s, annealing at 58 °C for 30 s, extension at 72 °C for 1 min, and final extension at 72 °C for 7 min. The recombinant transformers were digested with *Sal* I and *Bam*H I, and the products were separated on 1% agarose gel. The fragment was about 450 bp, indicating that *Math5* fragment had been inserted into the vector. Moreover, DNA sequencing was performed by using the primer specific for the human cytomegalovirus (CMV) immediate early promoter, located at the 5' end of the multiple cloning site (MCS). The result confirmed that the recombinant plasmid of *Math5* was constructed correctly.

### Cell transfection

Twenty-four h before transfection, cells were seeded into uncoated T25 flasks at a density of 1x10^6^ cells/ml with a total volume of 5 ml. Next, 5 μg of the recombinant pIRES2-EGFP-*Math5* was transfected into cells by using Lipofectamine 2000 (Invitrogen, Carlsbad, CA) according to the manufacturer's protocol. After 24 h incubation in transfection medium, some cells were collected for differentiation while, others were cultured in A culture medium (DMEM/F12 medium containing 4 mM L-glutamine, N2 supplement, B27 supplement, 20 ng/ml of bFGF, and 20 ng/ml of EGF) at 37 °C in 95% humidity and 5% CO_2_. Then cells were harvested for RNA isolation and reverse transcription-polymerase chain reaction (RT-PCR) after 48 h.

### Cell differentiation

RPCs were induced into differentiation under the same condition and divided into three groups: (A) RPCs transfected by pIRES2-EGFP-*Math5*; (B) RPCs transfected by pIRES2-EGFP; and (C) RPCs without transfection. Briefly, cells were collected and centrifuged at 1000 rpm for 5 min. After removal of the supernatant, cells were diluted to a concentration of 1x10^5^ in B culture medium, which was composed of Neurobasal^TM^-A medium (Gibco) containing 1xN2 supplement, 1xB27 supplement and the addition of 10 μM BrdU. Cells were seeded onto coated coverslips and cultured at 37 °C in 95% humidity and 5% CO_2_. After 24 h, fresh B culture medium without BrdU was used to replace the old medium. Cells were cultured for 10-14 days in all.

### Immunocytochemistry

We used the methods described by Hatakeyama, et al. to determine the proliferating nature and progenitor properties of cultured cells [6]. Cells cultured on coverslips or spheres attached onto coverslips were fixed in 4% paraformaldehyde (PFA) in 0.01M phosphate-buffered saline (PBS) for 15-20 min. Immunocytochemistry was carried out using standard protocols. Briefly, cells were permeabilized by treatment with 0.3% Triton X-100 for 30 min and 5% goat serum for 40-45 min and incubated in primary antibodies overnight at 4 °C. Primary antibodies used in this study were as follows: mouse anti-Nestin (neuroectodermal stem cell marker, 5.1 μg/ml; Chemicon, Temecula, CA), mouse anti-BrdU (proliferating cell marker, 11 μg/ml; Chemicon), mouse anti-Thy1.1 (ganglion cell-specific marker, 10 μg/ml; Biolegend, San Diego, CA) and rabbit anti-GS (Muller glia-specific marker, 2 μg/ml, Sigma). We used 0.01M PBS to replace primary antibodies in negative control. Then cells were incubated in CY3- or FITC-conjugated secondary antibodies for 2-3 h at 4 °C. Cells were counterstained with 5 mg/ml Hoechst33258 (Sigma) in some experiments for quantifying. The detection of BrdU required treatment in 4M HCl at 37 °C for 20 min. Images were taken by a fluorescence microscope (Leica DM IRB, Leica Microsystems GmbH, Wetzlar, Germany) equipped with phase-contrast optics or confocal laser scanning microscope (Leica TCS SP2, Leica Microsystems GmbH, Wetzlar, Germany) and analyzed by Leica Qwin V3.1 system. Positive cells were quantified in at least 10 fields systematically across the coverslips from three independent experiments of parallel cultures. All data were expressed as the mean±SEM. Statistical analysis was performed with Games-Howell in one-way ANOVA in SPSS11.5.

### Reverse transcription polymerase chain reaction and real-time polymerase chain reaction

Total cellular RNA of cells transfected by pIRES2-EGFP-*Math5* was isolated by using TRIZOL reagent (Invitrogen) according to the manufacturer's protocol. All cells were treated with *DNase*1, but only one tenth from 1x10^7^ cells were reverse-transcribed with random primers by superscript II (Invitrogen). To detect the expression of *Math5* mRNA, the same PCR amplification protocol as plasmid construction was performed, using the same oligonucleotide primer pair. The products were separated on 1% agarose gel. The positive control and negative control were separately obtained from pIRES2-EGFP-*Math5* and pIRES2-EGFP by amplification.

There were 18 total cellular RNAs isolated separately from three different groups on six time points (0 day, 1 day, 2 days, 4 days, 8 days, and 16 days after plating). To analyze the expression of Delta-1, Hes1 and Brn-3b genes, we performed real-time PCR (iCycler, Bio-Rad, Hercules, CA) amplifications using Taqman probes specific for the target genes. Cellular glyceraldehydes-3-phosphate dehydrogenase (GAPDH) mRNA from the same cell lysate was used as an internal control for cell number and metabolic status. The sequences of the primers and probes specific for the target genes are listed in Table 1. Next, 41 cycles of PCR were performed with cycling conditions of 3.5 min at 95 °C, 20 s at 94 °C, and 40 s at 58 °C. The real-time PCR signals were analyzed using iCycler iQ^TM^ software (Version 3.0). Each experiment was repeated at least three times.

**Table 1:** Sequences of the primers and probes for the target genes.

**Gene**	**Sense primer (5'-3')**	**Antisense primer (5'-3')**	**Probe**
Delta-1	GGGCTTCTCTGGCTTCAACTG	TTGCCGAGGTCCACACACT	Fam-TAGCTCTTCCCCTTGTTCTAACGGTGCC-Tamara
Hes1	CACAGAAAGTCATCAAAGCCTATCA	TTCTTAAGTGCATCCAAAATCAGTGT	Fam-AGAGGCGCCGGGCAAGAATAAATG-Tamara
Brn-3b	AGGCGATGCGGAGAGCTT	GCCAGCAGACTCTCATCCAGA	Fam-TCTTCCAACCCCACCGAGCAATATATTCG-Tamara

The sequences of the primers and probes specific for Delta-1, Hes1, and Brn-3b genes were used to analyze their expression.

## Results

### Cell culture

**Figure 1:**
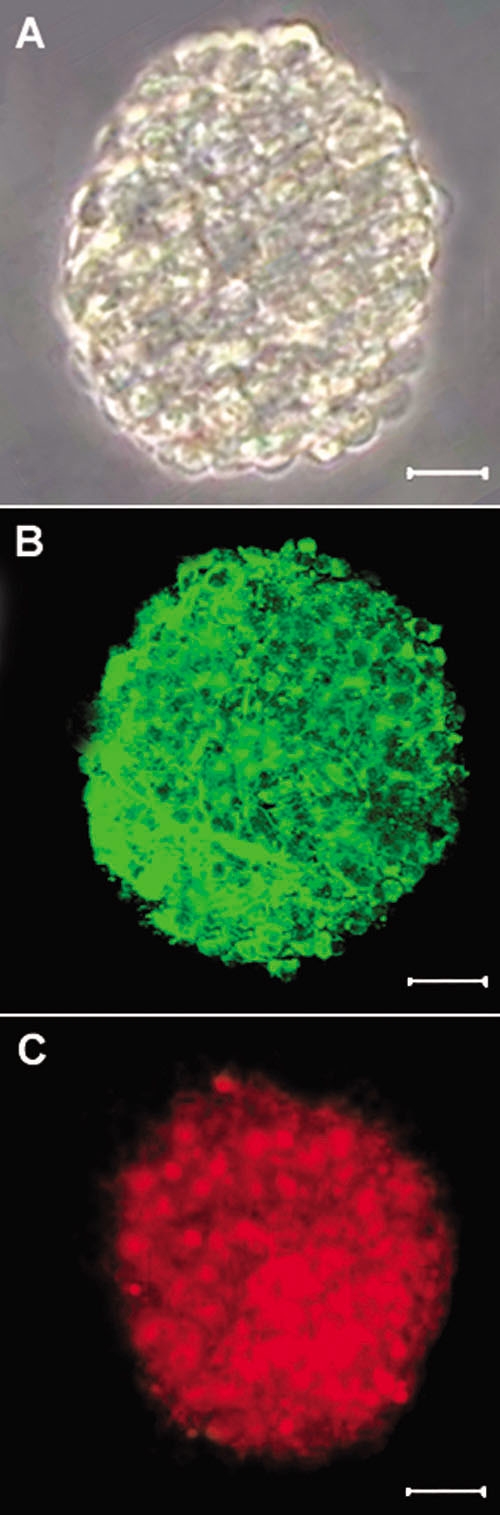
Culture of retinal progenitor cells. **A**: Phase-contrast micrograph of a neurosphere from passage 3, showing that it is composed of many cells. **B**: Most cells in the sphere prior to differentiation exhibited Nestin (green). **C**: Most cells in the sphere prior to differentiation exhibited BrdU (red). Scale bars equal 150 μm.

RPCs proliferated in the presence of EGF and bFGF and formed four-cell spheres after 2 days. The spheres increased gradually, and stable clonal spheres (Figure 1A) formed after 5 days. Most cells in spheres were immunopositive for Nestin, a marker for neuroectodermal stem cells (Figure 1B) and BrdU, a marker for proliferating cells (Figure 1C).

### Cell transfection

**Figure 2:**
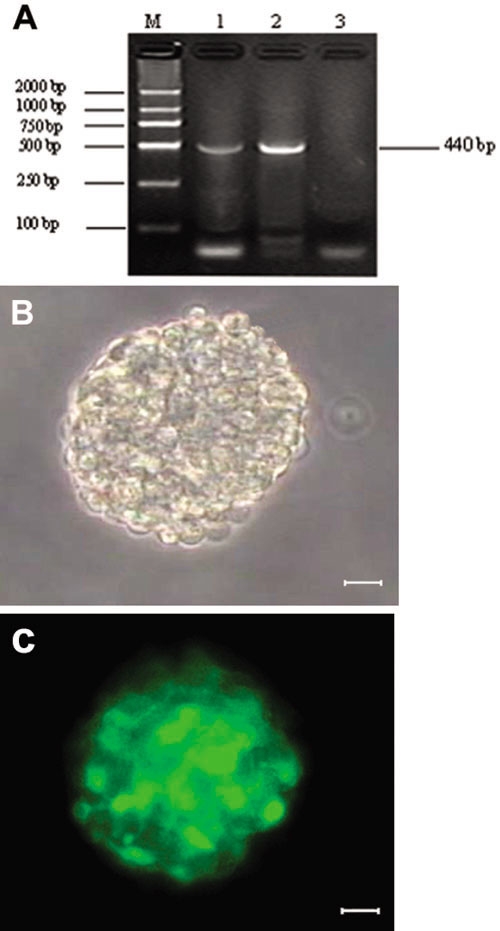
Cell transfection. **A**: RT-PCR product from cells transfected by pIRES2-EGFP-*Math5* was 440 bp. The row is labeled as follows: M (DL2000 DNA Marker); 1 (cells transfected by pIRES2-EGFP-*Math5*); 2 (positive control); and 3 (negative control). **B**, **C**: The same neurosphere was seen by phase-contrast microscopy (**B**) and fluorescence microscopy (**C**). A subset of cells expressed green fluorescence 48 h after transfection. Scale bars equal 150 μm.

Expression of *Math5* mRNA: After treatment with *DNase*1, residual genomic DNA and recombinant plasmid were both removed. Compared to the control, the specific fragment detected on 1% agarose gel was about 440 bp (Figure 2A), indicating that *Math5* mRNA was transcribed from pIRES2-EGFP-*Math5*.

Expression of enhanced green fluorescent protein: The pIRES2-EGFP vector contained the IRES of the encephalomyocarditis virus (ECMV) between the MCS and the EGFP coding region. The IRES element could be combined to cellular ribosome leading to translation which permit both the *Math5* and the EGFP gene to be translated from a single bicistronic mRNA. Therefore the expression of EGFP could represent that of *Math5*. Observations by fluorescence microscope showed that green fluorescence in the cytoplasm of RPCs could be clearly seen 24 h after transfection. The intensity of fluorescence declined gradually 48 h after transfection. The transfection rate was 24.68% (Figure 2B,C).

### Cell differentiation

**Figure 3:**
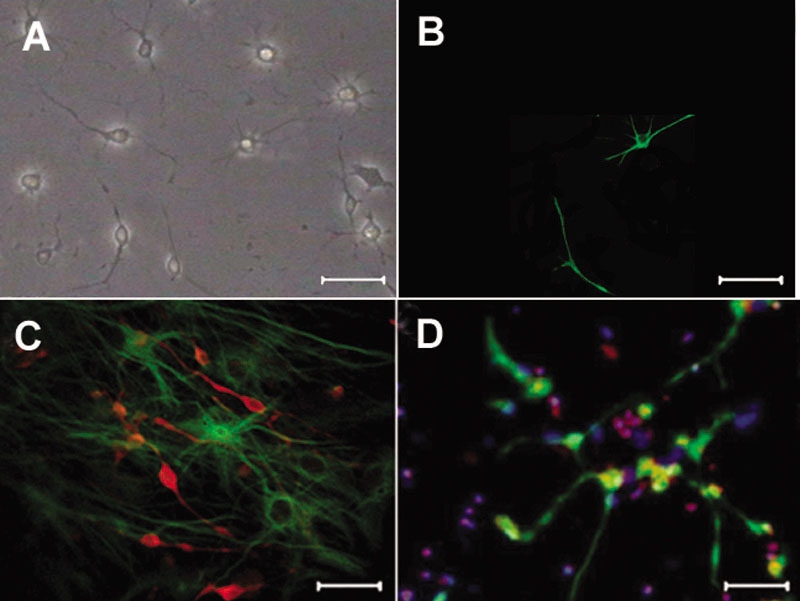
Retinal progenitor cells differentiation. **A**, **B**: The cells of group A were observed by phase-contrast microscopy (**A**) and fluorescence microscopy (**B**). Cells differentiated and spread processes 4 days after plating and green fluorescence was seen in the cytoplasm of some differentiated cells. **C**: A population of cells differentiated into Thy1.1 (red)- and GS (green)-immunopositive cells 2 weeks after plating. **D**: A population of RPCs differentiated into Thy1.1 (green)-immunopositive cells and a population of cells were still expressed BrdU (red) 2 weeks after plating, Hoechst (blue). Scale bars equal 75 μm.

The growth of RPCs in three different groups was similar. After plating, cells in the spheres migrated outwards and began to differentiate. Fluorescence microscope studies showed that cells migrated from spheres, spread processes and expressed GFP 1-2 days after plating, and fluorescence weakened gradually 7 days after plating and disappeared 14 days after plating (Figure 3A,B). Moreover, a subset of these cells could express retina-specific markers, including Thy1.1-positive RGCs and GS-positive Muller glia (Figure 3C). In order to investigate the influence of *Math5* on RGCs production and cell proliferation, Hoechst was used for quantifying (Figure 3D). It was found that the differences of the percentage of RPCs differentiating into RGCs were statistically significant (F=71.10, p<0.001). The percentage of RGCs in group A was higher than that of group B (t_A-B_=15.00, p<0.001) and group C (t_A-C_=14.63, p<0.001) and the differences of the percentage of RGCs between group B and group C were not statistically significant (t_B-C_=0.37, p=0.923). However, the differences of the percentage of proliferating cells were not statistically significant (F=0.02, p=0.980, Table 2).

**Table 2:** The percentage of RGCs(Thy1.1+/Hoechst) and proliferating cells(BrdU+/Hoechst) in three different groups.

**Group**	**RGCs %**	**F**	**P**	**Proliferating cells %**	**F**	**P**
A	30.85±6.28	71.1	<0.001	29.65±4.96	0.02	0.98
B	15.84±3.55			29.48±5.42		
C	16.22±3.60			29.75±5.50		

Comparison of the influence of Math5 on RGCs production(Thy1.1+/Hoechst) and cell proliferation(BrdU+/Hoechst) among three different groups.

### Real-time polymerase chain reaction

**Figure 4:**
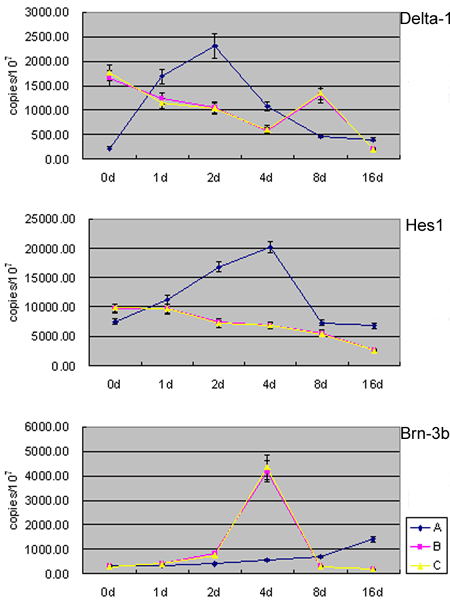
The expression of *Math5*-associated genes. Real-time polymerase chain reaction showed the expression of Delta-1, Hes1, and Brn-3b in three different groups, indicating that pIRES2-EGFP-*Math5* changed their expression.

The expression of *Math5*-associated genes, including Delta-1, Hes1, and Brn-3b was different on day 0, day 1, day 2, day 4, day 8, and day 16 during the differentiation of RPCs. In group A, the expression of Delta-1 and Hes1 increased progressively at first, submitted on the second day and fourth day separately, and then decreased progressively. The expression of Brn-3b increased gradually. In groups B and C, the expression of these genes was similar. The expression of Delta-1 decreased gradually before day 4 and began to increase. It finally submitted on day 8 and decreased again. Hes1 expression decreased gradually. Brn-3b expression increased slowly first, then shot between days 2 and 8, then it began to decrease slowly (Figure 4).

## Discussion

This study was designed to investigate the regulating role of *Math5* on RGC expression patterns in RPCs. We constructed recombinant pIRES2-EGFP-*Math5* and transfected cultured RPCs to make them over-express *Math5*. There were two major findings from this study: (1) Over-expression of *Math5* can promote RGC expression patterns in RPCs; and (2) over-expression of *Math5* could change the expression of *Math5*-associated genes.

During development of the mammalian retina, all retinal cells arise from a common progenitor with temporal and spatial precision [9]. In almost all species, RGCs are produced first. In rodent retina, RGCs emerged originally at E12, submitted at E14, continued toward E16, and then decreased significantly. bHLH transcription factors are responsible for the differentiation of RGCs. The role of *Math5*(Atoh7), a mouse bHLH gene, during early stages of retinal neurogenesis, was first reported by Dr. Brown [7]. It was expressed in the developing rodent retina starting at E11, preceding all other proneural genes, submitting at E13.5 and E14.5 to E16.5 and descending significantly afterwards. The spread of *Math5* expression from the dorsal cup circumferentially toward ventral matched the progression of RGCs layer formation. Given the highly conserved structure and expression patterns of *Math5* with atonal [10] in Drosophila, Ath5 [11] in zebrafish, Xath5 [12] in frog, and Cath5 [13,14] in chick, and the determination of atonal in formation of the first R8 neuron during Drosophila retinal development, we speculated that *Math5* was a good candidate for a proneural gene required for RGC formation. To investigate the role of *Math5* in retinal development and, particularly in the development of RGCs, we used recombinant plasmid to transfect RPCs. We found that the percentage of Thy1.1-positive RGCs was higher than those transfected by pIRES2-EGFP or those without transfection. The differences of the percentage of RGCs between those transfected by pIRES2-EGFP and those without transfection were not significant, indicating that transfection itself did not influence the differentiation of RPCs.

bHLH genes may exert their effects by influencing the proliferation as well as the differentiation of RPCs [5]. Our study demonstrated that the role of *Math5* on RPCs was primarily due to the differentiation of RPCs. We speculated that *Math5* might determine RGC competent state of RPCs after the terminal mitosis. Tracing the fate of Math5-expressing cells in developing retina demonstrated that Math5 expression was restricted to postmitotic cells and the loss of Math5 function severely reduced the expression of the transcription factors related to RGC differentiation, such as Brn-3b. However, *Math5* expression alone was not sufficient to determine the RGC fate [15], which implied that *Math5* expression was required to activate a comprehensive transcription network of RGC differentiation, and additional positive and negative factors were required in determining RGC fate. One likely explanation was that the prolonged expression of *Math5* in RPCs could increase the number of RGC competent progenitors, enable the progenitors to remain RGC competent for an extended period and more RGCs were generated. Alternatively, compared with the transient expression of endogenous *Math5*, the lasting, high level expression of exogenous *Math5* could overcome the negative regulation in the non-RGC pathway and lead to an increase in RGC differentiation.

The hierarchical gene regulatory network driving RPCs to differentiate into RGCs was complex and *Math5* occupied a key position in the hierarchy. The expression of *Math5* coincided with the onset of RGC differentiation and associated with the down-regulation of Notch-1. When ligand Delta from neighboring cells combined with Notch-1 receptor, anti-neural genes such as Hes group [16], were activated, which maintained RPCs, promoted their proliferation, and inhibited their differentiation. When the Notch pathway was down-regulated, proneural genes such as Ath group were activated. RPCs escaped neighboring inhibition, enhanced reactivity to proneural genes and started differentiation program [17]. *Math5* could activate Delta-1 expression [18], which inhibited differentiation in neighboring cells through activation of the Notch pathway, preventing all cells from differentiating simultaneously and adopting the same fate [19]. *Math5* mutant mice lacked RGCs and optic nerves. Targeted deletion of *Math5* abolished the retinal expression of Brn-3b in a dosage-sensitive manner and the formation of virtually all Brn-3b expressing RGCs, implying that *Math5* acted upstream to activate Brn-3b-dependent differentiation processes in RGCs [8,20-22]. Over-expression of Hes1 in embryonic retina inhibited neuronal differentiation and maintained progenitors. In Hes1 mutants, *Math5* expression was precocious along with RGC, but *Math5* expression was not up-regulated. It is possible that Hes1 inhibited the transcriptional activity of *Math5* by sequestering their cofactors [23]. The expression of Hes1 and *Math5* was Pax6-dependent in a dosage-sensitive manner. In Pax6 mutants, *Math5* expression was down-regulated and Hes1 expression was up-regulated. In a study of Hes1, Pax6 double mutants, and Hes1 mutants conducted by Lee et al., they demonstrated that Hes1 and *Math5* acted in opposite and independent manners [24]. But the regulating role of *Math5* on RGC differentiation was still poorly understood.

In the present study, real time PCR was used to examine the expression of *Math5*-associated genes at different time points during the differentiation of RPCs. It was found that the over-expression of *Math5* could change the expression of these genes. We speculated that *Math5* may interact with the genes associated with RGC expression patterns that resulted in more production of RGCs. The possible explanations were as follows: (1) Over-expression of exogenous *Math5* initiated the endogenous negative regulation resulting in progressive increase of Delta-1 and Hes1; (2) over-expression of exogenous *Math5* finally overrode the endogenous negative regulation resulting in progressive decrease of Delta-1 and Hes1 and; (3) over-expression of exogenous *Math5* may increase the RGC competent progenitors and prolong their period of differentiation which led to gradual increase of Brn-3b. However, the expression of Brn-3b in group A was lower than that in groups B and C, a finding that seemed to be inconsistent with the RGCs production. Two speculations need further studies: (1) Over-expression of exogenous *Math5* may trigger non-Brn-3b-dependent differentiation processes in RGCs pathway; and (2) more RGCs production may activate intrinsic regulatory mechanism, which prevented all cells from differentiating into RGCs by inhibiting the expression of Brn-3b.

Further studies to identify the key regulatory genes responsible for RGC differentiation with microarrays and to investigate the expression of the key regulatory genes in *Math5* disruption or over-expression models will contribute insights into neurogenesis.
